# An Experimental and Theoretical Analysis of Upstream Pumping Effect of Deep Spiral Grooves on Mechanical Face Seals

**DOI:** 10.3390/ma18122877

**Published:** 2025-06-18

**Authors:** Shaoxian Bai, Jiaqi Liu, Jing Yang

**Affiliations:** College of Mechanical Engineering, Zhejiang University of Technology, Hangzhou 310032, China; ljq13664211691@163.com (J.L.); yangjing@zjut.edu.cn (J.Y.)

**Keywords:** upstream pumping effect, deep groove, spiral face seals, experimental and theoretical analysis

## Abstract

The upstream pumping effect of mechanical face seals has a significant influence on their sealing performance. In order to reveal the effect of deep grooves on upstream pumping effects, an experimental and theoretical analysis is carried out in this study. The main novelty of this paper is to analyze the feasibility of deep grooves in a mechanical seal design from the perspective of cavitation and leakage rate. Firstly, an upstream pumping spiral groove is designed and fabricated, with different groove depths from 2 μm to 90 μm. Then, testing is performed with water as the sealing medium. Finally, the cavitation phenomena are captured, and leakage rates are measured during the experiment. The obtained results show that the groove with a depth of tens of microns can be designed according to the laminar flow hypothesis, and Reynolds equation is still valid to predict the cavitation and leakage rate theoretically. The spiral groove with a depth of tens of microns shows a significant upstream pumping effect. Both the theoretical and experimental analyses show that under certain working conditions, deep grooves can realize the zero-leakage sealing design of liquid, which might provide significant guidance for the sealing design of mechanical face seals to enhance sealing performance.

## 1. Introduction

The upstream pumping effect of grooves plays an important role in decreasing the leakage rate of mechanical face seals [1,2,3]. The effective structure design of surface grooves could enhance the upstream pumping effect and then achieve zero leakage performance [4], such as spiral grooves (by Salant in 1992 [5] and 1993 [6] and Brunetiere in 2021 [7]), straight grooves (by Lebeck in 2008 [8]), and elliptical grooves (by Bai in 2019 [9], Jiang in 2020 [10], and Liu in 2023 [11]). Zero leakage could even be achieved at a complex working condition of multi-speed ranging from 500 to 20,000 rpm and multi-seal pressure ranging from 0.1 to 3.0 MPa by proper design of surface grooves [12,13]. Generally, the depth of surface grooves is designed as shallow ones at the scale of a few or a dozen micrometers so as to obtain a significant upstream pumping effect as well as opening capacity. However, during the start–stop stage, contact wear is inevitable when fluid lubrication is not established, which often leads to the damage of surface grooves on sealing surfaces. With increasing demand for equipment reliability and service life, except for exploring improvements to wear resistance by coating on sealing surfaces (by Menga in 2019 [14]), the deep groove of tens to hundreds of microns is encouraged in the design to mitigate or avoid the adverse unexpected effects of sealing surface contact wear on the pumping effect.

Theoretically, the upstream pumping effect is induced by the shear effect of velocity on fluids in surface grooves, which is affected by the groove depth. Deep groove lubrication has always been a problem in lubrication analysis. In 2006, Feldman [15] discussed the validity of Reynolds equation in modeling the hydrostatic effects of pressure flow in gas-lubricated textured parallel surfaces with multi-pores of 15~20 μm depth in the cases of film thicknesses of 3~5 μm. Pressure distribution obtained from Reynolds equation was compared with that from the Navier-Stokes equations. It was illustrated that, even at large clearances of 5% of the dimple diameter and pressure ratios of 2.5, the error in the load carrying capacity is only about 15%. For a wide range of practical clearances and pressures, Reynolds equation can safely be applied to yield reasonable predictions for the load carrying capacity of grooved surfaces. In 2012, Bai’s theoretical analysis [16] showed that, compared with the commercial CFD code Fluent, Reynolds equation has sufficient accuracy for both pressure flow and shear flow in the gas-lubricating analysis of mechanical face seals with discontinuous clearance on sliding surfaces, especially for shallow grooves at a scale of ten micrometers, although a significant recirculation zone comes out in the groove center with shear velocity increasing from 10 m/s to 100 m/s. But, for millimeter-scale deeper groove surfaces, the prediction of Reynolds equation often presents a large deviation. In 2015, Ding’s experimental analysis [17] of the gas hydrostatic lubrication of rectangular deep grooved surfaces illustrated that there is a significant throttling effect induced by millimeter-scale deep grooves of 0.1~1.5 mm, which decreases the leakage rate obviously. There is a significant deviation between the experimental value of the gas leakage rate and the theoretical value obtained from the Reynolds equation after the flow state transfers from a laminar to a turbulent state. When the Reynolds number is less than 3000, the experimental value of the leakage rate is relatively close to the theoretical value. When the Reynolds number is greater than 3000, the gap between the theoretical results and the experimental results increases sharply with an increase in the Reynolds number. The theoretical values obtained from the Reynolds are much larger than the experimental. CFD analyses by Sahlin [18] and Guardino [19] indicate that deep grooves on surfaces have a significant impact on fluid flow, and large-scale vortices will occur within the grooves, which often leads to a reduction in the leakage rate. However, for the upstream pumping-related shear flow, the effect of deep grooves is still not very clear, especially at a scale of tens of microns deep.

In particular, for liquid-lubricated face seals, cavitation is often generated in the grooves by shear action, which affects and restricts the upstream pumping effect [20,21,22]. To some extent, the size of the cavitation zone reflects the strength of the upstream pumping effect. In 2011, Qiu [23] carried out experimental research into oil-lubricated cavitation on the surfaces of circular and elliptical holes with depths of 50~60 microns. The results show that the occurrence and development of cavitation are significantly affected by rotational speed. When the rotational speed is lower than 40 rpm, no cavitation zone is generated in the liquid film. When the rotational speed is 40–800 rpm, the cavitation zone increases significantly with an increase in rotational speed. When the speed is higher than 800 rpm, the bubbles in the cavitation zone burst and cause disturbance. It seems that the rotational speed causes changes in the fluid flow state in the deep groove of tens of micrometers. However, it is still not very clear how the rotational speed affects the upstream pumping performance of face seals as the groove depth increases from a few micrometers to tens and hundreds of micrometers.

This study intends to explore the feasibility of achieving zero leakage by deep groove upstream pumping so as to further expand the range of groove depths in the design of face seals. An experimental test is carried out on the upstream pumping effect of deep spiral groove face seals. Cavitation is observed, and the leakage rate is measured for different depth grooves at different speeds and seal pressures. The validity of Reynolds equation is discussed to predict the cavitation and leakage rate.

## 2. Experimental Part

**Figure 1:**
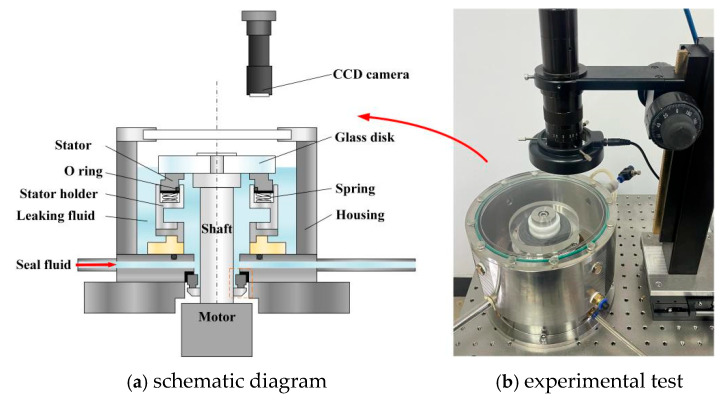
Visual device for sealing test: (**a**) schematic diagram and (**b**) experimental test.

Figure 1 shows the set-up of an upstream pumping spiral groove face seal consisting of a rotor and a stator. The rotor ring is made of K9 glass, which is fixed into a metallic holder on the shaft and driven by a motor with rotational speed controlled by a computer. The stator material is carbon graphite on which face spiral grooves are fabricated. In order to compare the result between the experimental and numerical methods, the materials of the rotor and the stator are the same. The high-pressure seal fluid is fed into the inside of the stator ring. The cavitation phenomenon in the grooves is observed and captured by a CCD camera. Meanwhile, leaking fluid is collected in the housing and measured after three-minute testing.

High-pressure water, as seal fluid, is fed into the inner diameter side of the seal rings through a connecting hole at the bottom of the housing. When the upper glass disk starts rotating, the lower stator ring moves a few micrometers along the axial direction, forming a clearance between the glass disk and the lower ring. Some high-pressure water flows from the inner diameter side to the outer through the clearance due to high pressure at the inner diameter side, forming a leakage rate of the face seal, which is measured as the leakage rate.

**Figure 2:**
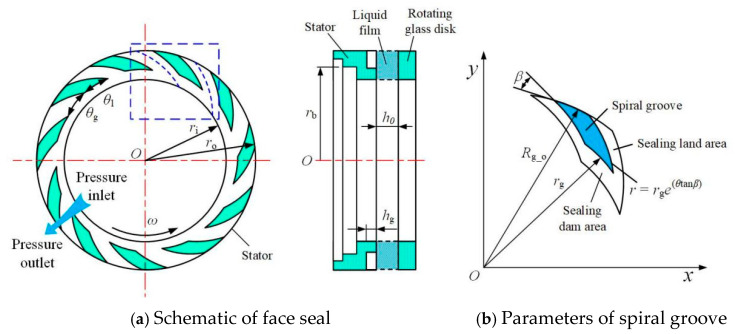
Schematic diagram of spiral groove face seal: (**a**) schematic of face seal with periodical simulation domain (the blue dashed box), and (**b**) parameters of spiral groove.

**Figure 3:**
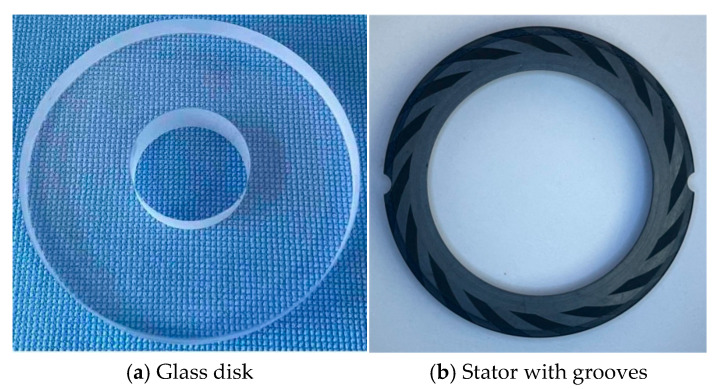
Testing samplers: (**a**) glass disk and (**b**) stator with grooves.

A schematic description of the upstream pumping spiral groove seal rings is presented in Figure 2. In order to capture the cavitation image more conveniently, the spiral groove is machined on the static rings. The testing seal rings are shown in Figure 3, and upstream pumping spiral grooves are fabricated on the stator faces, as shown in Figure 3b. The main structure parameters of the testing seals are given in Table 1.

**Figure 4:**
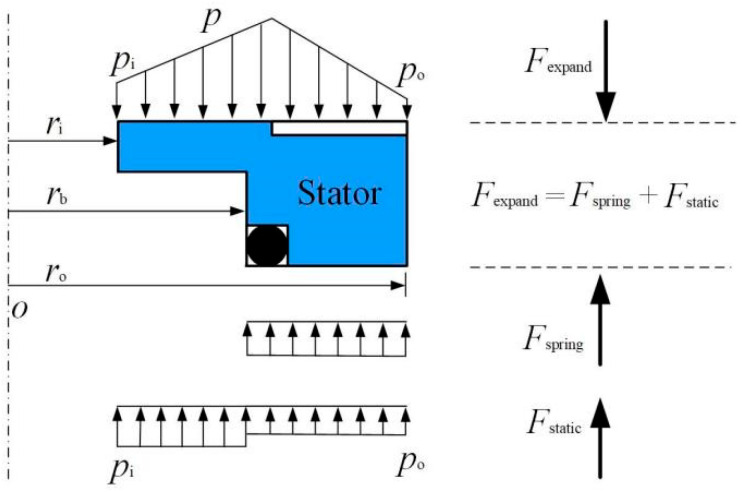
Force balance diagram acting on the seal ring.

During the test, under the action of rotational speed, the hydrodynamic pressure of the spiral grooves causes a fluid lubricating film to form between the glass disk and the stator ring, driving the stator ring to move axially for a certain distance to form a non-contact sealing gap. When stable, the static ring is in a state of force equilibrium, as shown in Figure 4.

The pressure at the outside and inside of the sealing rings is *p*_o_ and *p*_i_, respectively. They could correspond to a balance between the gas film opening force *F*_expand_ and static closing force *F*_pack_. The closing force *F*_pack_ is equal to the sum of spring force *F*_spring_ and static pressure closing force *F*_static_. The following equation would be obtained when the seal structure is in a steady running state:

(1) $$F_{\mathrm{expand}}=F_{\mathrm{pack}}$$

The opening force can be given as

(2) $$F_{\mathrm{expand}}=\int_{0}^{2\pi }\int_{r_{i}}^{r_{o}}pr\;drd\;\theta$$

The closing force can be expressed as

(3) $$F_{\mathrm{pack}}=F_{\mathrm{static}}+F_{\mathrm{spring}}=\pi \left(r_{o}^{2}-r_{b}^{2}\right)p_{o}+\pi \left(r_{b}^{2}-r_{i}^{2}\right)p_{i}+F_{\mathrm{spring}}$$

In this experimental study, the spring force *F*_spring_ is set as 20 N, which is applied to the stator.

For each working condition, the test was repeated three times, and the leakage rate was taken as the average of the three test measurements. The error was taken as the average error of three measurements.

## 3. Theoretical Model

The theoretical model for the upstream pumping effect of mechanical face seals has been developed and described in previous work. Here, the cavitation effect is added to make the simulation results more precise. The predominantly used governing equations, cavitation definitions, boundary conditions, element meshing, and analysis parameters can be summarized as follows.

### 3.1. Reynolds Equation

In the analysis of mechanical face seals, Reynolds equation is commonly applied as the main equation of theoretical models to solve the pressure distribution [24]. Its polar coordinate form could be expressed as

(4) $$\frac{1}{r}\frac{\partial }{\partial r}\left(\frac{\rho rh^{3}}{\eta }\frac{\partial p}{\partial r}\right)+\frac{1}{r}\frac{\partial }{\partial \theta }\left(\frac{\rho h^{3}}{\eta }\frac{\partial p}{r\partial \theta }\right)=6\omega \frac{\partial \rho h}{\partial \theta }$$

where *ρ* is the density, *ω* is the circumferential speed, *p* is the local pressure, *η* is the viscosity of the medium, and *r* and *θ* are the polar coordinates.

### 3.2. Cavitation

The compressibility of fluids in lubrication theory is mainly represented by density variation. In this study, we assumed that the fluid in the cavitation region is in a complete gas state. The cavitation pressure *p*_c_ is the critical pressure at which the phase state of the sealing medium transfers from liquid to gas, while the density is equaled to the density *ρ*_0_ in the liquid state. Therefore, the cavitation pressure of gas in the cavitation region should satisfy the following equation:

(5) $$\frac{\rho }{p}=\frac{\rho_{0}}{p_{c}}\;\;p\leq p_{c}$$

In the liquid lubrication analysis, the density of gas in the cavitation region could be described as the following:

(6) $$\left\{\begin{array}{cc} \rho =\rho_{0} & \begin{array}{cc} \mathrm{if} & p>p_{c} \end{array}\\ \frac{\rho }{p}=\frac{\rho_{0}}{p_{c}} & \begin{array}{cc} \mathrm{if} & p\leq p_{c} \end{array} \end{array}\right.$$

Since the sealing medium at the low-pressure side of the mechanical face seal is air during testing, the value of critical cavitation pressure is set to 0.1 MPa in the following numerical analysis.

### 3.3. Boundary Conditions

**Figure 5:**
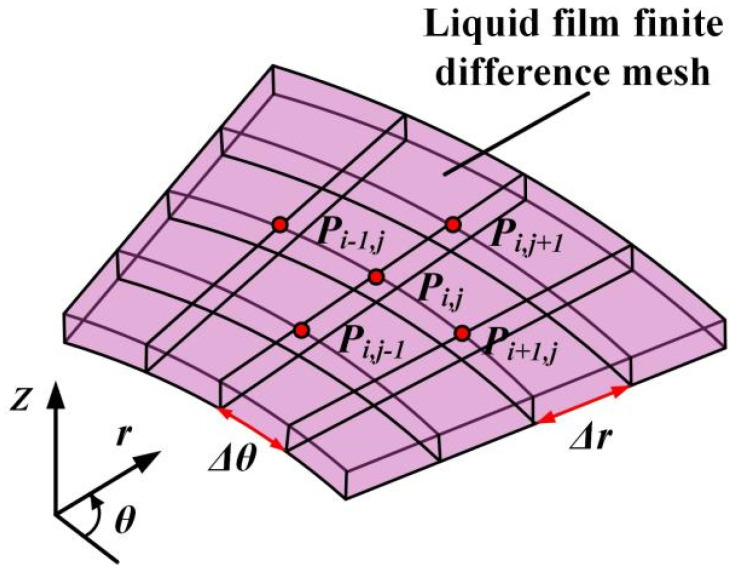
The periodic calculation domain.

Since the spiral grooves were distributed evenly along the circumferential direction, the periodic domain is selected as the numerical simulation region, as shown in Figure 5, which can efficiently reduce computational time. The pressure boundary conditions based on the periodic calculation region could be defined as the following:

(7a) $$p(r=r_{i},\theta )=p_{i}$$

(7b) $$p(r=r_{o},\theta )=p_{o}$$

(7c) p(r,θ=π/N)=p(r,θ=−π/N)

### 3.4. Numerical Method

Detailed information on this numerical calculation can be found in the work of [13]. Using the same numerical method, the pressure distribution, opening force, and leakage rate can be obtained. The equation of leakage rate can be expressed as

(8) $$q=\frac{1}{12\eta }\int_{0}^{2\pi }h^{3}r\frac{\partial p}{\partial r}d\theta$$

The convergence criterion is defined as

(9) $$\delta p=\left|\frac{p^{k}-p^{k/2}}{p^{k}}\right|$$

where *k* is an iterative number.

## 4. Cavitation Effect

In our published works, we have discussed the validity of the above cavitation model by comparing it with the experimental works of Etsion [25] and Zhang [26]. Generally, cavitation brings about a positive effect in the lubrication analysis of mechanical dynamic components [27,28,29,30]. In order to investigate the cavitation change influenced by groove depth, the cavitation phenomenon is observed by the experimental method. Here, further comparison work is carried out to discuss the cavitation for deeper grooves of a dozen micrometers.

**Figure 6:**
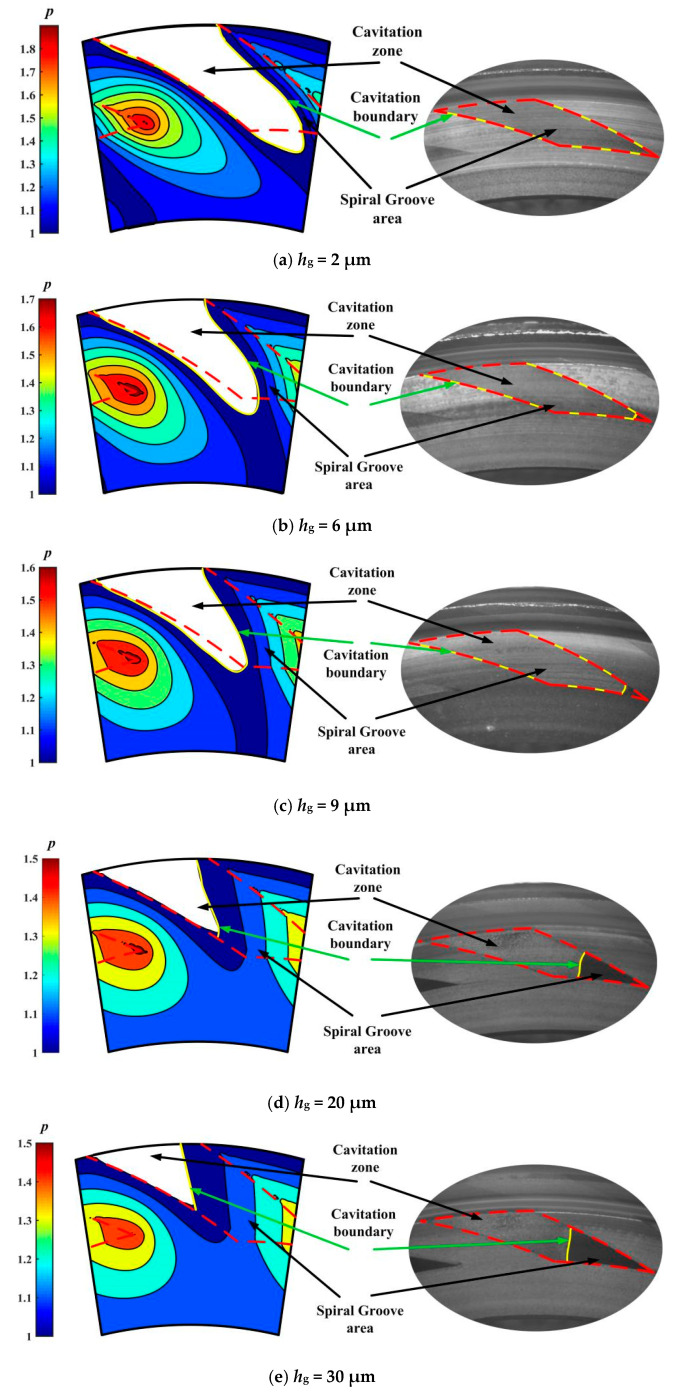

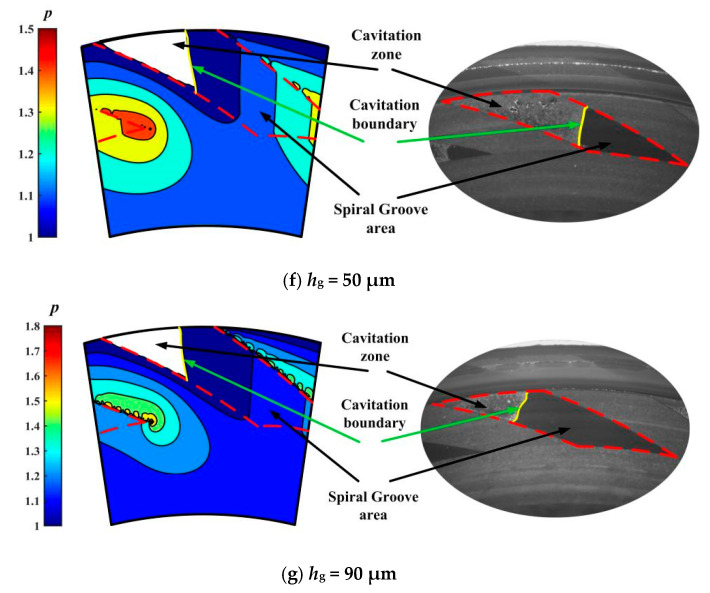
Comparison of cavitation region captured in experiment with that obtained from theoretical model (*w* = 2000 rpm, *p*_i_ = 0.11 MPa): (**a**) *h*_g_ = 2 μm, (**b**) *h*_g_ = 6 μm, (**c**) *h*_g_ = 9 μm, (**d**) *h*_g_ = 20 μm, (**e**) *h*_g_ = 30 μm, (**f**) *h*_g_ = 50 μm and (**g**) *h*_g_ = 90 μm.

Figure 6 illustrates the comparison of the cavitation region captured in the experiment with that obtained from this theoretical model. It can be seen that there is obvious cavitation in the grooves under shear action of speed. For the 2 μm depth grooves, the entire groove area is almost completely cavitation. Moreover, the theoretical and experimental cavitation zone sizes show the same trend with the increase in groove depth. In theory, cavitation in the lubricating film is caused by the negative pressure generated by velocity shear in the groove area. Generally, the higher the speed and the smaller the groove depth, the higher the shear rate of the fluid, resulting in a smaller pressure in the negative pressure zone, a larger area, and a larger cavitation area. So, as expected based on lubrication theory, with increasing groove depth from 2 μm to 90 μm, the cavitation zone size decreases obviously in the test. This means that the depth of tens of microns is still applicable to the laminar flow hypothesis to some extent, and Reynolds equation is still valid in the prediction of cavitation.

More importantly, for the 90 μm depth grooves, cavitation still occurs in about 25% of the groove area. This means that the upstream pumping effect may also be significant in deeper grooves of tens of microns. Since the size of the cavitation zone reflects to some extent the strength of the shear flow in the lubrication zone, it can be concluded that a 90-micron deep groove can also produce an obvious upstream pumping effect and a hydrodynamic effect.

## 5. Upstream Pumping Effect

The upstream pumping effect is a flow along the groove from the low-pressure side to the high-pressure side under shear action of rotational speed, which is limited by groove depth and sealing pressure to a large extent. In this section, the influence of groove depth and seal pressure on cavitation and the leakage rate is discussed.

### 5.1. Groove Depth

According to the laminar flow assumption of hydrodynamic lubrication theory, no matter how deep the groove is, there is a pumping effect brought on by shear flow, and as long as the shear velocity is large enough, cavitation may often occur in the grooves. Zhao [28]’s research into spiral groove face seals shows that the cavitation rate increases with an increase in rotational speed, and it rises by more than six times with speed increasing from 1000 to 4000 rpm.

**Figure 7:**
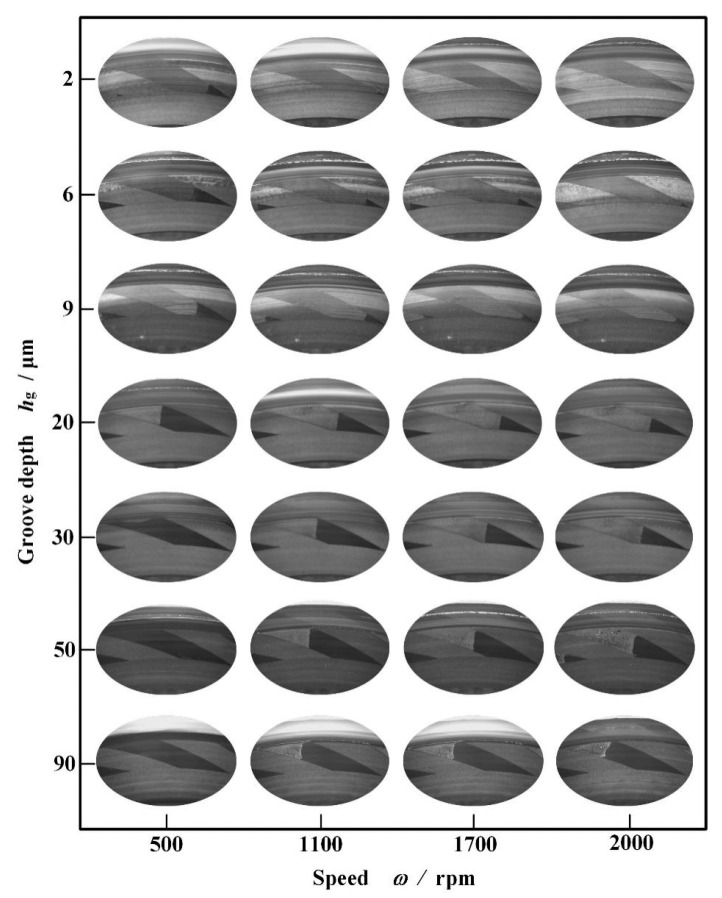
Cavitation in the grooves of different depths at different speeds (*p*_i_ = 0.2 MPa).

Figure 7 illustrates cavitation in grooves of different depths at different speeds in this test. It can be seen that, as expected in theory, there comes an obvious whole cavitation area at the inlet of the grooves even when the groove depth exceeds 30 μm. Meanwhile, with increasing speed, the cavitation area becomes larger. This means that deeper grooves also present a significant pumping effect.

The reason is that the shear effect of speed takes more fluid away in deeper grooves, although the shear ratio becomes smaller there theoretically. This is also consistent with the laminar flow hypothesis in the derivation of Reynolds equation.

**Figure 8:**
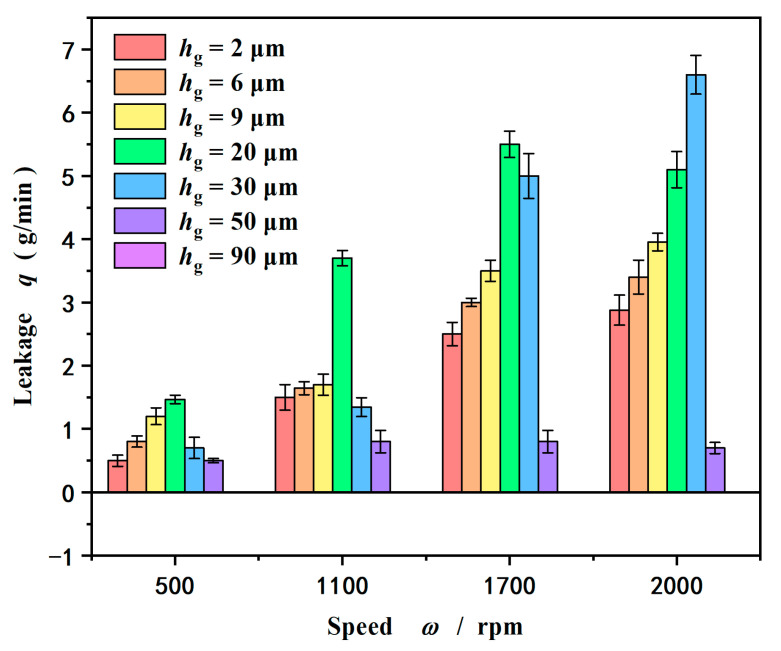
Influence of groove depth on leakage rate at different speeds (*p*_i_ = 0.2 MPa).

Figure 8 gives the influence of groove depth on leakage rate at different speeds. On the whole, as expected, the leakage rate increases with an increase in speed. The reason is that the spiral groove produces a significant hydrodynamic effect, making a larger seal clearance, which leads to a larger positive leakage rate induced by seal pressure flow. On the other hand, the larger clearance makes the upstream pumping effect weak due to the lower shear rate. So, in the case of a seal pressure of 0.2 MPa, the values of the leakage rate are positive. As pointed out by Salant [6], appropriate groove parameter values are required to achieve zero-seal leakage control. This may become more complex for deep-depth grooves.

More importantly, as shown in Figure 8, the leakage rate does not always increase with increasing groove depth. The leakage rate reaches a maximum value with increasing groove depth and then drops. At a speed of 1100 rpm, the leakage rate reaches a maximum value of 6 × 10^−8^ m^3^/s at a groove depth of 30 μm. When the speed increases to 1700 rpm, the leakage rate reaches a maximum value of 9 × 10^−8^ m^3^/s also at a groove depth of 30 μm. Further, when the speed increases to 2000 rpm, the leakage rate reaches a maximum value of 11 × 10^−8^ m^3^/s also at a groove depth of 50 μm and then decreases, even zero at a groove depth of 90 μm.

There are two reasons for the above nonlinear variation in the leakage rate with the depth of the groove. One is that the hydrodynamic pressure effect begins to decrease when the groove depth increases to a certain value, resulting in a reduction in the sealing clearance. Because the pressure flow is highly sensitive to the size of the sealing gap, the leakage rate decreases. Another is the throttling effect produced by the deep groove.

**Figure 9:**
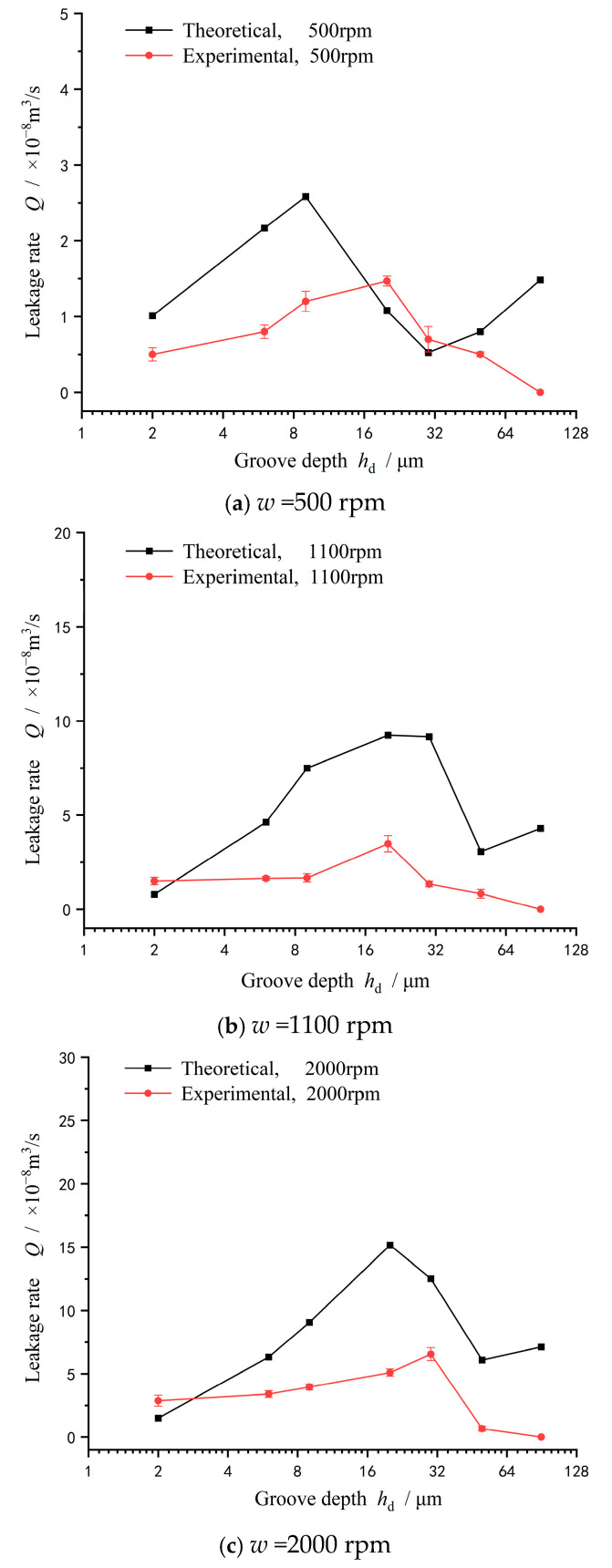
Theoretical prediction of leakage rate at different speeds (*p*_i_ = 0.2 MPa): (**a**) *w* =500 rpm, (**b**) *w* =1100 rpm, and (**c**) *w* =2000 rpm.

In order to further analyze the reason causing a leakage decrease at deep groove surfaces, Figure 9 gives the theoretical prediction of the leakage rate compared with the experimental. Obviously, the theoretical and experimental leakage rates show the same trend with the increase in groove depth before the groove depth exceeds 50 μm. With increasing groove depth from 2 μm to 50 μm, the leakage rate increases to a peak value at about a groove depth of 30 μm and then decreases. The theoretical and experimental deviation of the leakage rate is about 15% at a groove depth of 30 μm.

It should also be noted that when the groove depth is larger than 50 μm, the numerical values of the leakage rate obtained from the theoretical model begin to deviate from the experimental, becoming larger than the experimental with an increase in groove depth, especially at a lower speed of 500 rpm. The deep groove throttling effect can better explain this phenomenon. According to Ding’s experiment [17], the groove with a depth of 0.1 mm coupled with a 0.3 mm clearance also produces a significant throttling effect, making the flow rate decrease by about 10~20%. Meanwhile, Reynolds number has no direct relationship with the throttling effect, and surface deep grooves can still cause a 30% decrease in the flow rate at a low Reynolds number of 345 when a completely turbulent flow state has not been reached. Sahlin [18] and Guardino [19] attributed this throttling effect to the vortex flow within the groove through CFD analysis.

### 5.2. Seal Pressure

Seal pressure is another parameter affecting cavitation and upstream pumping effects. Generally, the higher the sealing pressure, the smaller the cavitation zone and the weaker the upstream pumping effect.

**Figure 10:**
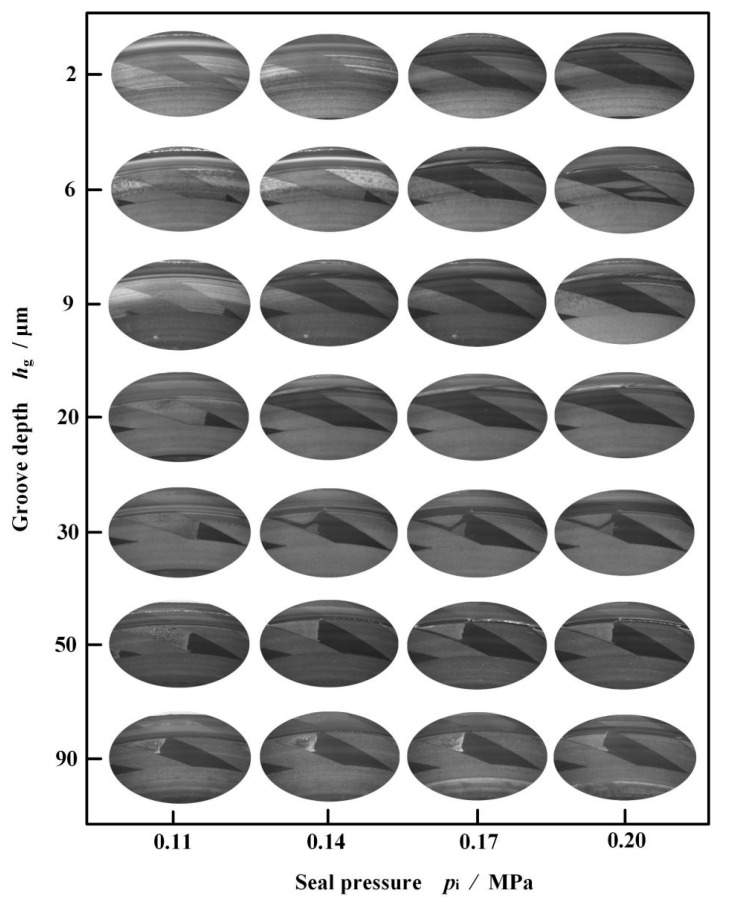
Cavitation in the grooves of different depths at different seal pressures (*w* = 2000 rpm).

Figure 10 illustrates cavitation in the grooves of different depths at different seal pressures at a speed of 2000 rpm. It is obvious that the size of the cavitation area in the grooves decreases with an increase in both seal pressure and groove depth. The reason is that as the seal pressure increases, more liquid flows into the groove to replenish the fluid that is sheared away by the speed, and the cavitation area decreases. In addition, the increase in groove depth is conducive to the inflow of fluid, which further reduces the occurrence of cavitation.

**Figure 11:**
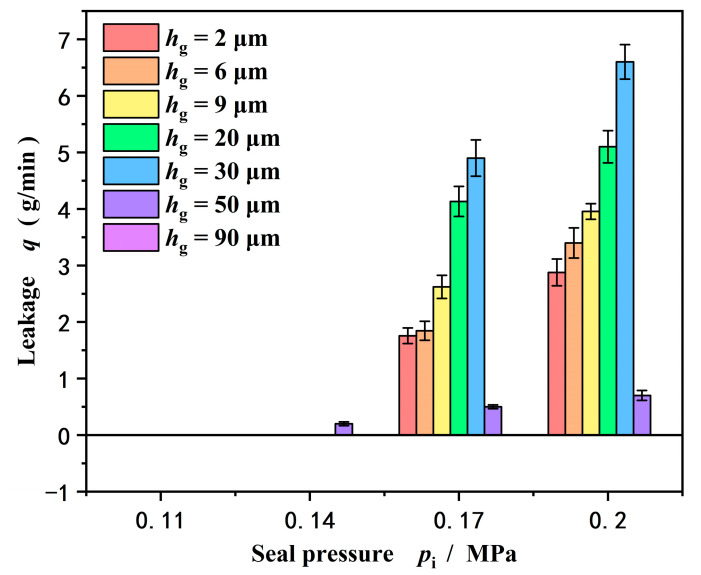
Influence of groove depth on leakage rate with increase in seal pressure (*w* = 2000 rpm).

Figure 11 gives the influence of groove depth on leakage rate with an increase in seal pressure at speed *w* = 2000 rpm. On the whole, as expected, the leakage rate increases with an increase in seal pressure but not groove depth. The leakage rate reaches a maximum value with increasing groove depth and then drops, as discussed above.

Another important result is that the leakage rate measured in the test is almost zero at seal pressures of 0.11 MPa and 0.14 MPa. The reason for this phenomenon is that under the condition of lower sealing pressure, the seal forms a completely upstream pumping with zero leakage. Meanwhile, the leakage also present a zero value at a deep groove depth of 90 μm as discussed above. That is, in the experiment, no water leaked out from the inner diameter to the outer diameter under high pressure.

**Figure 12:**
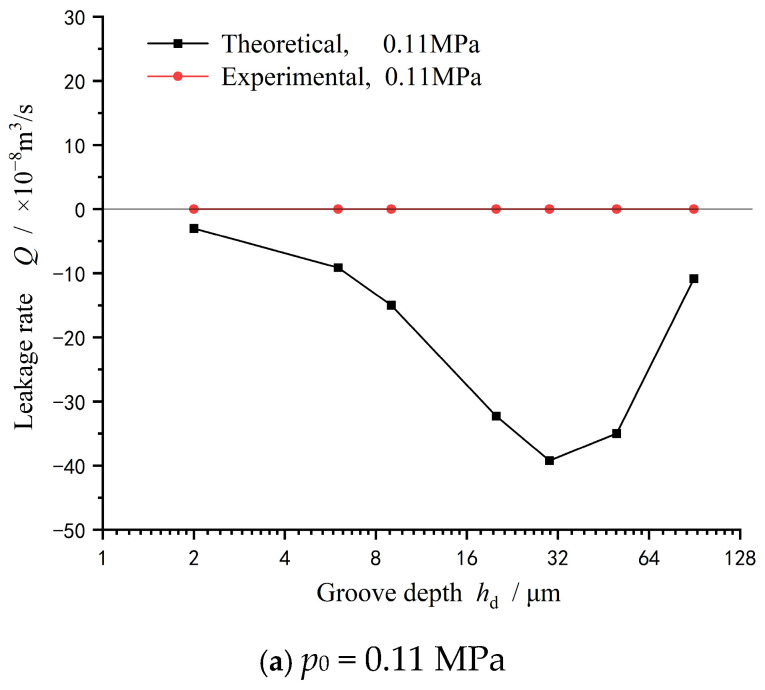

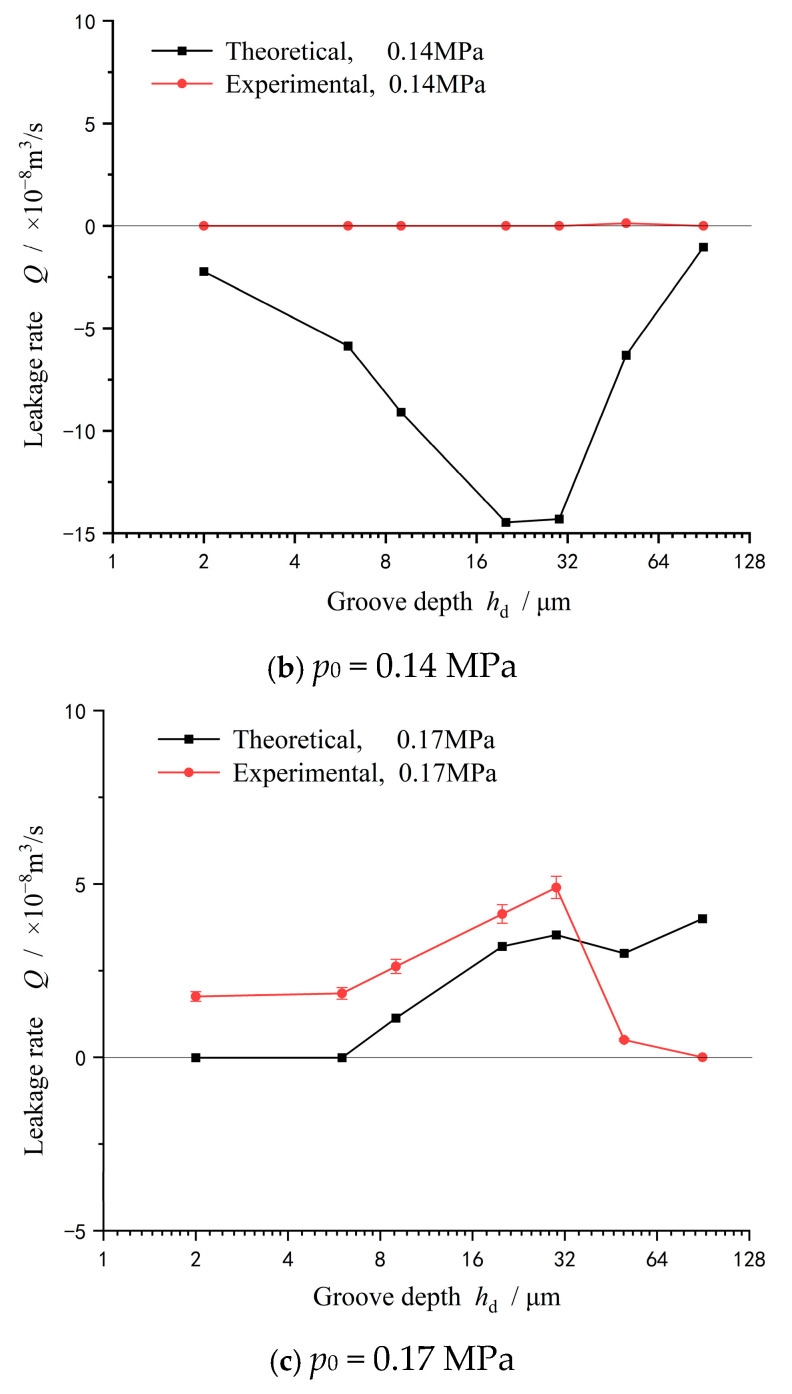
Theoretical prediction of leakage rate at different seal pressures (*w* = 2000 rpm): (**a**) *p*_0_ = 0.11 MPa, (**b**) *p*_0_ = 0.14 MPa, and (**c**) *p*_0_ = 0.17 MPa.

In order to further discuss the validity of the theoretical model, Figure 12 gives the theoretical prediction of leakage rate at different seal pressures. Obviously, under the lower seal pressure conditions of 0.11 MPa and 0.14 MPa, the seal achieves a full upstream pumping state, and the leakage rate measured in the experiment is 0, while the theoretical prediction of the leakage rate is negative. In other words, the theoretical model can predict 0 leakage for sealing, which is consistent with the experimental test.

Moreover, when the seal pressure is further increased to 0.17 MPa, the theoretical prediction and test measurement show the same trend of the leakage rate increasing first and then decreasing.

## 6. Conclusions

In this study, a numerical model was used to calculate the upstream pumping effect and the cavitation effect of mechanical face seals with spiral grooves. In order to achieve zero leakage performance, a deep groove structure was designed according to the laminar flow hypothesis. The cavitation phenomena were captured, and leakage rates were measured during the experimental test. Based on the numerical and experimental results, the following conclusions could be drawn:

(a) Under lower seal pressure conditions of 0.11 MPa and 0.14 MPa, the mechanical seal could achieve a full upstream pumping state, and the leakage rate was 0 according to the experimental test, while the theoretical prediction of the leakage rate at the same condition was negative. When the sealing pressure increased to 0.17 MPa, the theoretical prediction and test measurement of leakage rate showed the same trend, increasing first and then decreasing.
(b) The spiral groove with a depth of tens of microns showed a significant upstream pumping effect. Both theoretical and experimental analyses showed that under certain working conditions, deep grooves could realize the zero-leakage sealing design of liquid.
(c) Though the theoretical and experimental analyses of this study were aimed at spiral grooves, the obtained results could also provide guidance for the depth design of other surface grooves, which could be beneficial to future sealing design in engineering applications.

Here, changes in the fluid flow state were not considered in the theoretical model, resulting in certain limitations still existing in the established theoretical model. Judging from the experimental results, it is relatively accurate for analyzing cavitation and pressure distribution. However, when the groove depth exceeds 50 μm, the leakage volume results given by the theoretical model show an obvious deviation. In future research into deeper grooves, especially in theoretical modeling, flow states such as turbulence and vortices may need to be taken into account.

## Figures and Tables

**Table 1:** Structure parameters of the testing seals.

Parameter	Symbol	Value
Outer radius	*r*_o_/mm	35
Inner radius	*r*_i_/mm	27
Outer groove radius	*R*_g_o_/mm	35
Inside groove radius	*r*_g_/mm	31
Spiral angle	*β*/°	24
Balance radius	*r*_b_/mm	30
Groove number	*Z*	16
Slot area circumference angle	*θ_l_*/°	11.25
Table area circumference angle	*θ_g_*/°	11.25
Liquid film thickness	*h_o_*/μm	
Groove depth	*h_g_*/μm	2–90
Seal speed	*ω*/rpm	500–2000

## Data Availability

The original contributions presented in this study are included in the article. Further inquiries can be directed to the corresponding author.
